# What Shall We Do with Inebriates?

**Published:** 1881-05

**Authors:** 


### WHAT SHALL WE DO WITH INEBRIATES?

In an able article on this subject in the *Alienist and Neurologist*, Dr. Crother, Superintendent of the Walnut Hill House, reaches the following conclusions:

1. Inebriety is a physical disease with a distinct origin, development and progress. Its symptomology is continuous and can be traced from stage to stage.

2. In the causation the desire for alcohol is both a symptom and a disease. Different effects come from different alcohols, and different degrees of functional and organic perversions complicate and enter into the causation. Inebriates are divided into classes which require special methods and means of treatment.

3. When inebriety is studied as a special disease in hospitals for this purpose, its curability will be found equal to any other disease, and the answer to the question, What can be done for the inebriate? will be understood and practically carried out.

